# The Intraoperative Golden Hour in Minimally Invasive Parafascicular Surgery for Brain Tumors

**DOI:** 10.3390/cancers18081241

**Published:** 2026-04-14

**Authors:** José Pedro Lavrador, Yasir A. Chowdhury, Filippo Andrea Sinosi, Francesco Marchi, Vindhya Prasad, Oktay Genel, Ana Mirallave-Pescador, Alba Diaz-Baamonde, Richard Gullan, Keyoumars Ashkan, Francesco Vergani, Ranjeev Bhangoo

**Affiliations:** 1 Department of Neurosurgery, King’s College Hospital NHS Foundation Trust, London SE5 9RS, UKfrancesco.marchi@eoc.ch (F.M.); vindhya.prasad@nhs.net (V.P.);; 2 Neurology Department, Neurocenter of Southern Switzerland EOC, 6900 Lugano, Ticino, Switzerland; 3 Department of Neurosurgery, Cambridge University Hospitals NHS Foundation Trust, Cambridge CB2 0QQ, UK; 4 Department of Neurophysiology, King’s College Hospital NHS Foundation Trust, London SE5 9RS, UK

**Keywords:** minimally invasive parafascicular approach, tubular retractor, neuro-oncology, tractography, neuromonitoring, golden hour, craniotomy

## Abstract

Minimally invasive parafascicular surgery (MIPS) represents a paradigm shift in the management of deep-seated brain tumors, enabling function-sparing resections previously limited to biopsy and/or medical therapy. Central to MIPS are structured frameworks guiding preoperative planning and intraoperative execution. The six-pillar concept—comprising imaging, navigation, atraumatic access, optics, resection, and postoperative care—provides a comprehensive approach to integrate advanced neuroimaging, tractography, tubular retractor systems, fluorescence-guided resection, and neuromonitoring to optimize functional outcomes. Five-point target-trajectory complex planning—craniotomy, outer radial corridor, inner radial corridor, target, and resection margins—translates preoperative imaging and functional mapping into a precise surgical trajectory, balancing maximal tumor resection with minimal disruption of eloquent brain structures. Preoperative assessment of tumor characteristics, vascular relationships, and cortical eloquence informs trajectory planning and intraoperative adjustments. A critical determinant of MIPS success is the *intraoperative golden hour*, referring to the high-risk period surrounding brain cannulation with a tubular retractor. Key principles include (1) precannulation system checks to ensure instrument readiness; (2) access injury prevention through optimized craniotomy sizing and sulcal preparation; (3) tubular-tumor targeting accuracy addressing brain and tubular translation, tumor displacement, and white-matter sleeves; and (4) intracranial pressure control strategies to minimize tissue strain and venous congestion. Overcoming this period enables a controlled resection phase guided by the above-mentioned surgical adjuncts. The *six-pillar concept* and *five-point target-trajectory complex planning* are the foundations of MIPS planning, whereas the *intraoperative golden hour* provides a roadmap for successful intraoperative delivery of the surgical plan.

## Simple Summary

Some brain tumors are located deep within the brain, close to important areas that control movement, speech, or vision. In the past, these tumors were often considered too risky to remove and were treated only with biopsies or non-surgical therapies. Minimally invasive parafascicular surgery (MIPS) is a newer surgical approach that allows surgeons to reach these deep tumors more safely by gently separating natural pathways in the brain rather than cutting through healthy tissue. This paper describes structured frameworks that help surgeons plan and perform MIPS safely. These include careful use of advanced brain scans, navigation systems, monitoring of brain function during surgery, and specialized tubular instruments. This study also highlights a critical stage during surgery the “intraoperative golden hour” when a careful technique is essential to prevent injury and control brain pressure. Together, these approaches aim to maximize tumor removal while protecting brain function, helping patients recover faster and with fewer complications.

## 1. Introduction

Minimally invasive parafascicular surgery (MIPS) is leading to a paradigm shift in the surgical management of challenging brain tumors. Deep-seated lesions previously managed with diagnostic procedures only (biopsy) or with medical oncology strategies (radiotherapy modalities and/or chemotherapy) are now amenable for surgical resection [1,2,3,4]. Furthermore, previous approaches to tumors requiring significant resection of non-involved tissue resection can now be accessed via tissue-sparing approaches with the potential for better functional outcomes translated into a decreased length of stay and complications [5,6,7].

MIPS has come a long way in the last decades since the first reports in the 1980s [8]. It has capitalized on the improved understanding of functional neuroanatomy alongside significant technical developments in neuro-navigation. In the last decade, a renewed interest into its use for intracerebral hematoma (ICH) management has disseminated its use in neurosurgical units [9]. Its application to neuro-oncology followed the knowledge acquired with ICH management and evolved into the nuances of tumor management in narrow surgical corridors [10]. Previous publications addressed the overall brain tumor treatment concepts—the six-pillar concept [11]—and detailed preoperative planning to optimize the MIPS technique−five-point target-trajectory complex planning [12]—in neuro-oncology. However, the intraoperative framework leading to a successful execution of presurgical planning and its underlying challenges and nuances have not been previously explored. In this manuscript, we will briefly review the previous published framework and elaborate on a proposed intraoperative stepwise approach for a successful MIPS procedure—the intraoperative golden hour for MIPS in neuro-oncology.

## 2. Minimally Invasive Parafascicular Surgery: The Six-Pillar Concept

**Figure 1:**
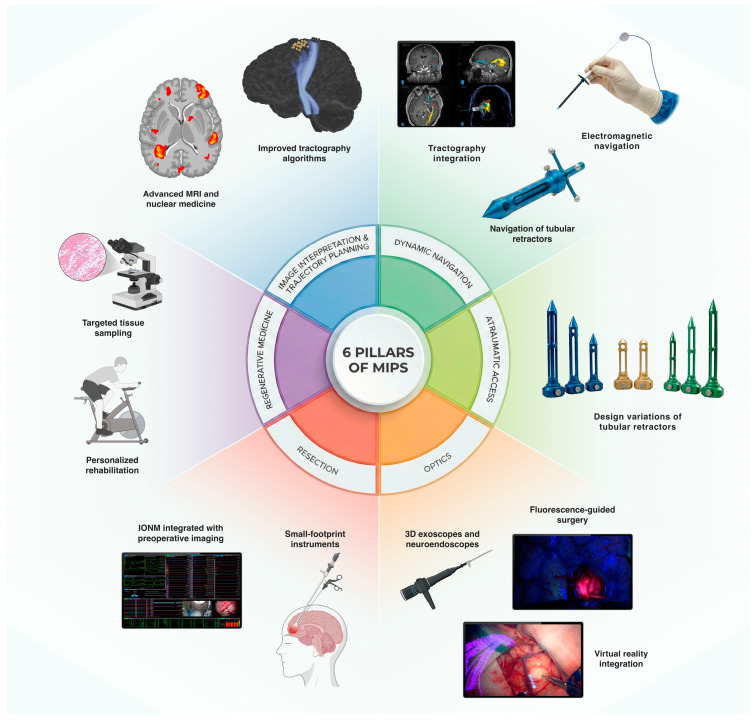
The six-pillar approach. (IONM: Intraoperative neuromonitoring).

The six-pillar approach, originally proposed by Ritsma et al., outlines the key procedural stages and technologies integral to MIPS [11]. These include (1) image interpretation and trajectory planning, (2) dynamic navigation, (3) atraumatic access, (4) optics, (5) resection, and (6) regenerative medicine, as summarized by Figure 1. More than a decade later, these pillars remain central, though significant evolution has occurred within each domain.

### 2.1. Image Interpretation

This has benefited from advances in nuclear medicine and MRI techniques, improving characterization of tumor infiltration and microenvironment [13]. Trajectory planning has been refined through enhanced image acquisition and improved algorithms that enable broader use of tractography in neurosurgical planning. This has supported more accurate, function-sparing approaches and is increasingly incorporated into risk stratification models integrating functional and structural connectivity [14,15,16].

### 2.2. Navigation Systems

Navigation systems have advanced with the integration of electromagnetic navigation within tubular retractor systems, object-based navigation of the tubular retractors themselves, and implementation of tractography algorithms directly within surgical navigation platforms [17].

### 2.3. Atraumatic Access

In atraumatic access, widespread adoption of tubular retractor systems has generated numerous comparative studies evaluating how design variations influence surgical access and patient outcomes [6,10].

### 2.4. Optics

Optics have expanded dramatically, with the integration of tract and tumor segmentation with navigated virtual reality into surgical microscopes [13]. The rise of 3D exoscopes and the incorporation of neuro-endoscopy into port-based approaches have transformed intraoperative visualization [18,19,20,21]. Fluorescence-guided surgery, delivered via microscopes, exoscopes, endoscopes, or in situ light sources, now bridges the domains of optics and resection by enhancing the extent of resection and supporting oncological outcomes [22].

### 2.5. Resection

Resection has been further optimized through intraoperative neuromonitoring integrated with preoperative functional and structural mapping, enabling safer function-preserving tumor removal. Electrified, small-footprint instruments suitable for tubular corridors allow real-time neurophysiological confirmation without compromising the visual field [23,24,25].

### 2.6. Regenerative Medicine

Finally, the regenerative medicine/personalized postoperative care pillar has broadened. Structural preservation achieved through MIPS facilitates functional recovery when paired with appropriate rehabilitation [26]. Simultaneously, targeted tissue sampling—uniquely enabled by minimally disruptive access—supports personalized post-resection oncological strategies [27,28,29]. Figure 1 summarizes the six-pillar concept.

## 3. Minimally Invasive Parafascicular Surgery: Five-Point Target-Trajectory Complex Planning

The careful planning of the cortical entry point and relationship to the subcortical anatomy are supported by the extensive literature supporting the preservation of preoperatively defined cortical and subcortical eloquent areas using navigated transcranial magnetic stimulation [15,30,31,32], functional MRI and tractography [14,33]. These techniques are often combined by teams around the world in risk stratification algorithms that assist in making treatment decisions. Also, in particular, cavity-to-tract approaches facilitate the avoidance of higher-functional-risk areas whilst planning trajectories to deep-seated lesions [34,35].

The five-point target-trajectory complex planning—craniotomy, outer radial corridor, inner radial corridor, target and resection margins—was defined to materialize the preoperative and intraoperative planning and surgical resection during the MIPS approach [12]. Even though it is enounced from the surface to the depth, the definition of each of these points follows an inverse order—depth to surface.

The first step in surgical planning is to understand the characteristics of the target, particularly its vascularity and density based on preoperative structural MRI sequences such as T2 and diffusion-weighted images [12,36]. It is also crucial to understand the vascular relationship with main vessels and perforator arteries based on preoperative vascular imaging. The above characteristics will define the docking target—as a rule of thumb in tumors, surface docking is preferred to allow for dissection and given the likelihood of higher cellular lesions with potential bleeding—and make a bridge for the resection margin step where the vascular relations are integrated with the preoperative structural planning provided by tractography algorithms. The resection margins are preoperatively planned and then established with intraoperative neuromonitoring and mapping [7].

**Figure 2:**
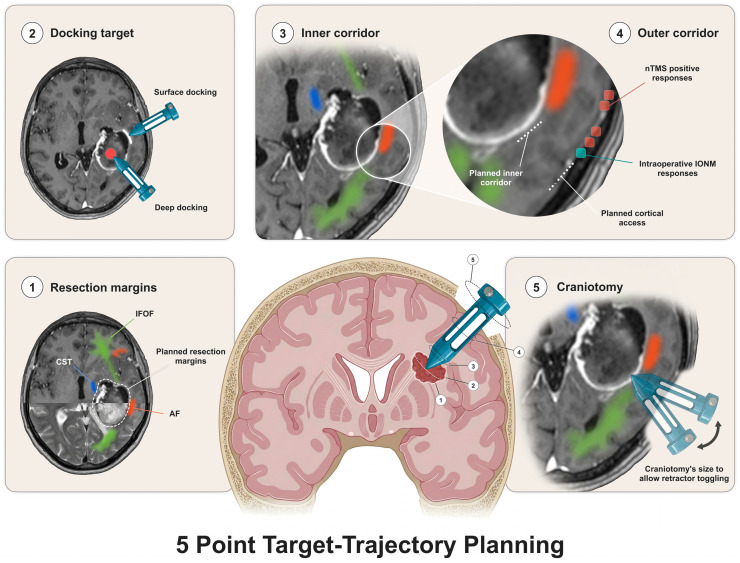
The five-point target-trajectory planning. (nTMS: navigated transcranial magnetic stimulation, IFOF: inferior fronto-occipital fasciculus, AF: arcuate fasciculus, CST: corticospinal tract).

Once the target is planned, an optimal function-sparing trajectory is defined, avoiding projection, commissural and the main association tracts (even though short association tracts may be disrupted given their close relationship with the sulci) with a favorable sulcal entry point [37]. The cortical eloquence is defined using functional cortical techniques such as navigated transcranial magnetic stimulation [38] or functional MRI [39]. Therefore, both the inner white matter and the outer cortical surface, radial corridors are defined simultaneously. This trajectory can be corrected intraoperatively with intraoperative neuromonitoring data related to cortical functional mapping using both high-frequency or low-frequency techniques and continuous monitoring offered by subdural cortical strips for motor and visual evoked potentials, for example [40]. Lastly, the craniotomy should be defined according to both outer and inner radial corridors, allowing for sufficient anticipated toggling of the tubular retractor according to the characteristics of the target and resection margins (Figure 2).

## 4. Minimally Invasive Parafascicular Surgery: Intraoperative Golden Hour

The *intraoperative golden hour* is not bounded to the timeframe implied in the literal expression but refers to the concept of a critical time period related to surgical stepwise procedures that are crucial for a successful MIPS-based tumor resection. In MIPS, tubular cannulation is the critical step in surgery. Regardless of the design and port size, a tubular retractor introduction always produces an increase in the intracranial pressure and functional–structural displacement of both tumoral and normal anatomy with the potential for either mechanical or vascular injury [5]. These can have consequences not only limited to the intracranial compartment, but also systemic repercussions related to pressure changes, such as Cushing response-like behavior with a raise in blood pressure and decrease in heart rate. Therefore, brain cannulation can be compared to a storm in the context of the MIPS-based tumor resection, where the pericannulation period is critical for overall surgical success. In summary, *the golden hour* is not a time-locked period, but a phase-dependent approach anchored to the pericannulation period that is unique and defining in MIPS.

Here, we discuss a *four-step surgical concept* crucial to overcome the critical period of brain cannulation to access a brain tumor.

### 4.1. Precannulation System Check

The brain cannulation induces a sudden change in the intracranial homeostasis. It causes an increase in the intracranial brain pressure, shear stress in the brain tissue parenchyma around the tubular retractor and potential tissue and vascular damage during the process. Therefore, it is crucial that the correct functioning of all equipment is checked before the brain cannulation is performed—this is the precannulation system check. Particular attention should be given to the following:

(1) *Correct placement of the holding devices* to secure the tubular retractor, making sure the planned cannulation can be done without the instruments being caught in skin retractors or other devices and so they do not obstruct visualization down the corridor.
(2) *Debulking/resecting instruments* are working properly and ready to use if required during/after cannulation. This is relevant as they are usually different from the instruments used until this stage of surgery either because of length considerations (different bipolar diameters and configurations) or de novo instruments altogether (micro-debrider instrument and ultrasonic aspirator). Finally, if personalized adjustments to the instruments are to be done, they should happen before cannulation to minimize the cannulation time (such as electrification or navigation of surgical instruments) [25].

**Figure 3:**
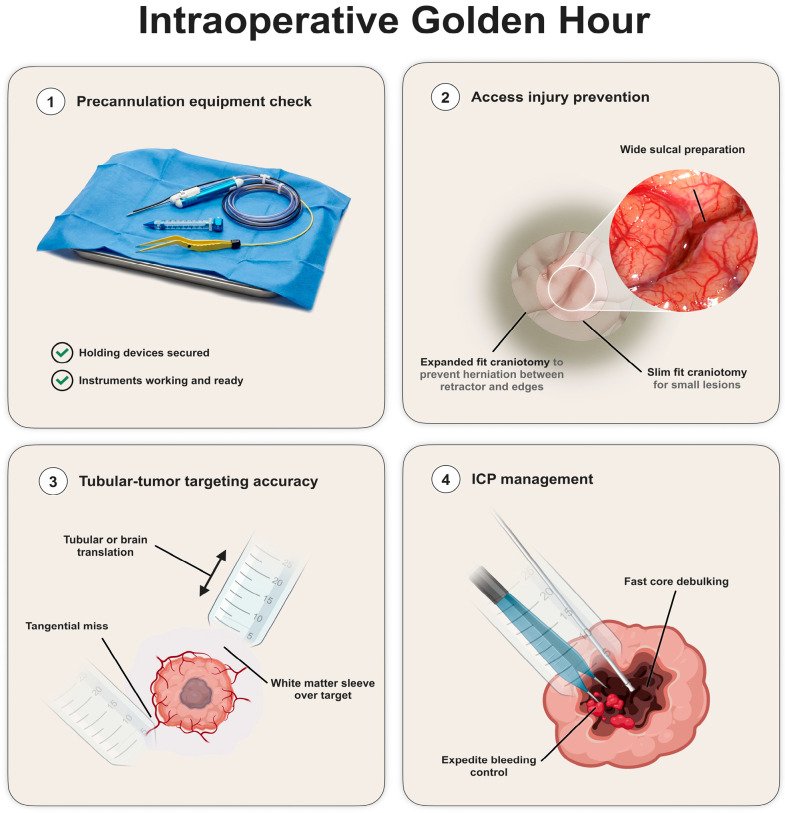
The four steps during intraoperative golden hour. (ICP: intracranial pressure).

Take into consideration that the above-mentioned steps prevent delays in addressing the tumor or complications arising during brain cannulation (Figure 3).

### 4.2. Access Injury Management and Prevention

Access injury is a concern for every neurosurgeon, which is intimately related with the core concept of the MIPS technique—function sparing and tissue preservation. If a significant injury is made during brain cannulation, the whole principle of MIPS is defeated. Also, minimizing access injury is related to better neurological outcomes [6] and a decreased length of stay [7] and therefore supports the significance of access injury prevention.

Multiple techniques have been described to avoid access injury related both with craniotomy size and sulcal preparation prior to cannulation [10]. The main concept related to craniotomy size is focused on the brain herniation between the tubular retractor and the edge of the craniotomy [17]. If present, this herniation can lead to direct tissue damage in the edges of both the tubular retractor and the bone edge. Also, it can lead to venous stasis due to kinking of venous structures in both above-mentioned structures, leading to a vicious cycle creating further herniation. Therefore, the craniotomy size should fall onto either a slim-fit size—large enough to accommodate only the tubular retractor—or an expanded fit where it is substantially larger that the tubular retractor diameter, to prevent damaging brain herniation between the tubular retractor and the bone edge.

Both approaches have pros and cons that should be tailored to each case. A slim-fit-size craniotomy will minimize the exposed brain and, in theory, the surgical time and infection risk. However, it will limit the amount of toggling allowed to the tubular retractor, causing less trajectory-related flexibility and limiting the use of other surgical adjuncts adjacent to the tubular retractor, such as intraoperative neuromonitoring strips for cortical monitoring and paratubular intraoperative ultrasound. Also, if the craniotomy is not precisely centered to a sulcus or it encounters a large vascular structure, alternative cannulation options will be limited. On the other hand, an expanded-fit craniotomy will lead to a larger incision and brain exposure, which, alongside the time and infection risks mentioned above, may entail a higher risk of tissue injury during dural opening. However, it provides more options for cannulation adjusted to intraoperative findings, and the use of regular cortical monitoring tools and ultrasound and allows an easier conversion for a conventional craniotomy if required.

In summary, the decision between a slim-fit and expanded-fit craniotomy is related to the surgical preference and personalized to the lesion being treated. Slim-fit approaches may be suitable for small lesions with cannulations along the long axis of the lesion in non-eloquent areas, whereas expanded-fit approaches may be more suitable for larger lesions in eloquent regions were a main lesional axis cannot be defined, therefore requiring an increased amount of tubular toggling. Related with craniotomy-based exposure is the concept of sulcal preparation/dissection prior to cannulation. Even though introduction devices are designed to minimize tissue and vascular damage, allowing for vascular displacement within the sulcus, a wider sulcal dissection with release of the arachnoid around the arteries and mobilization of veins from arachnoid attachments reduces tension within the sulcus induced by tubular cannulation and therefore the risk of vascular avulsion (arteries) and tearing (veins) (Figure 3).

In summary, an appropriate craniotomy and sulcal dissection work together to minimize access injury during brain cannulation.

### 4.3. Tubular-Tumor Targeting Accuracy

This surgical moment refers to the actual brain cannulation procedure and the concepts supporting its planning. As mentioned above, the default mode of cannulation for tumors is surface docking, except for highly necrotic soft lesions in highly eloquent areas where internal decompression may be the main surgical goal alongside tissue diagnosis. Often, after brain cannulation, when the obturator device is removed and the microscope provides visualization of the bottom of the tubular-retractor surgical corridor, there is a mismatch in between the information provided by the intraoperative navigation system (tumor or tumor margin) and the tissue visualized (normal-looking white matter). This is a critical moment that requires efficient and systematic troubleshooting given the sudden increase in the intracranial pressure induced by the introduction of the tubular retractor. This tubular-tumor targeting accuracy problem is related to the static nature of preoperative-based navigation systems in the face of a dynamic intraoperative maneuver of brain cannulation with a tubular retractor. Some surgical steps can provide a framework to address this inaccuracy:

(1) Tubular-Brain Translation during Cannulation: Two types of translation movements occur during brain cannulation: brain translation and tubular translation. The first one refers to the movement away from the skull along the axis of the cannulation produced by the tubular introduction against the brain. This displaces the target deeper in the direction of the cannulation and, even though it cannot be completely prevented, it can be minimized by performing a larger sulcal split to minimize the resistance during the tubular introduction. The tubular translation relates to the tubular displacement during removal of the obturator. This can produce a tubular dislocation in both directions along the axis of the cannulation. Surgical experience may play an important role in minimizing this second translation movement. Overall, tubular-brain translation most often produces a displacement of the target (tumor) away from the docking site along the cannulation axis.
(2) White-Matter Sleeve over the Target: Two mechanisms are responsible for the interposition of a sleeve of white-matter tissue in between the docking site and the target (tumor). One has been described above (tubular translation). The second is related to the transient negative pressure created by the removal of the tubular obturator, which displaces white-matter tissue inside the tubular retractor over the lesion. This tissue sleeve creates the illusion of an inaccurate targeting that must be overcome by its resection before encountering the tumor.
(3) Tumor Displacement: This is related with the triangulation between docking target, tumor consistency and the angle between the cannulation axis and the tumor. It has been mentioned above how preoperative imaging has aimed to inform the surgical team about tumor consistency and why surface docking is preferable for tumor resection. In a situation where the tumor is firm and our docking is beyond the surface of the lesion, displacement away from the surgical corridor can happen, particularly with smaller lesions. Also, if the angulation between the tumor and the cannulation axis deviates significantly from an orthogonal angle, there is a higher chance of lesional displacement away from the surgical corridor during the cannulation—a tangential effect. This displacement can be addressed via a systematic inspection of all the corridor quadrants, progressive retraction of the tube along the cannulation axis to allow for normalization of the tumor location in the case of a deep docking strategy, or utilization of intraoperative ultrasound down the tubular retractor (if available) or in a paratubular position if an expanded-fit craniotomy has been performed.
(4) Inappropriate Cannulation due to Limitation of the Craniotomy: Even though less common, this highlights the need for consideration of the target and the cannulation axis (inner radial corridor) for craniotomy planning and not only the outer radial corridor (cortical/sulcal entry point) so the tubular retractor can be introduced along the planned trajectory without being limited by the edges of the craniotomy. This is particularly relevant if a slim-fit craniotomy is planned.

Intraoperative imaging adjuncts can be used to assess and correct the tubular-tumor targeting accuracy providing real-time information over a static preoperative image acquisition. Intraoperative ultrasound can be used adjacent to the tubular retractor in expanded-fit craniotomies or down the tubular retractor with the appropriate probes to assess the relative position of the tubular retractor–tumor complex. We have used intraoperative CT and subsequent fusion with preoperative MRI for this purpose [41].

Intraoperative MRI is potentially feasible if the appropriate MRI-compatible holding system is available. Even though all these intraoperative imaging tools are potentially useful, intraoperative ultrasound is particularly appealing given its versatility and dynamic assessment as it allows for real-time visualization of tubular-tumor position correction [42].

### 4.4. Intracranial Pressure Control Strategy

This is the critical moment in tumor resection for a successful MIPS-based tumor resection. As soon as targeting accuracy is established, good control or the artificially induced decrease in the intracranial pressure is essential to minimize tissue damaged as described above, minimize non-involved white-matter tissue to herniate into the surgical field, and decrease venous congestion, which may increase intratumoral bleeding during the resection. Safer functional and oncological areas should be identified for a judicious debulking at the beginning of surgery to achieve these goals. The steps to begin surgical resection should be undertaken with no unnecessary delays. This should take precedence over tissue collection or further assessments as it is time-dependent. Ideally, it should target the core of the tumor as, regardless of the etiology, it is a safer functional area to address. After initial cannulation, further rebound of intracranial pressure can occur with intratumoral bleeding, particularly if it is away from the tubular retractor (perceived by brain swelling at the surface). This is the rationale for expedited bleeding control in these surgeries. Failure to achieve this pressure control can potentially compromise the whole MIPS-based tumor resection. In our experience, this was the main cause for conversion from the MIPS procedure to conventional craniotomy.

Based on the above four-step approach, we propose an intraoperative checklist to potentially increase the safety and streamline the intraoperative pericannulation setup (Table 1), However, this stepwise approach is not suitable for all clinical scenarios, particularly in the presence of a raised intracranial pressure. Here, the need to control the intracranial pressure with tumor debulking of CSF drainage may lead to a priori decision, such as expanded-fit craniotomy and less time dedicated to sulcal preparation given the transcalvarial parenchymal herniation—a transgyral approach instead of a transsulcal approach should be considered in these circumstances given the vascular risk or a poor sulcal split. Therefore, the tumor characteristics may determine variations to the above-presented golden hour approach.

## 5. Minimally Invasive Parafascicular Surgery: After Storm, Clarity and Focusing on Resection Challenge

**Figure 4:**
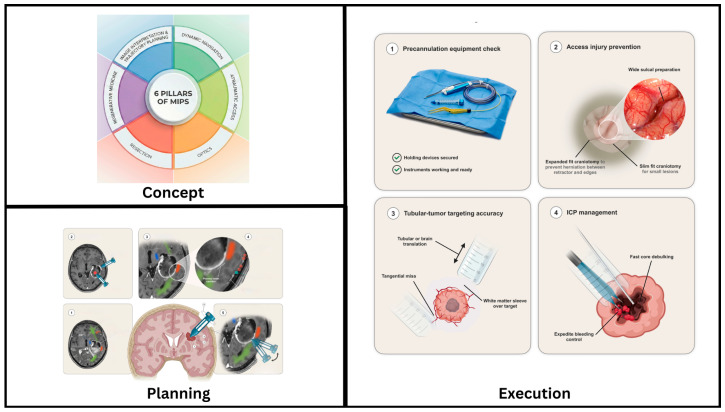
Summary of MIPS approach technique: Concept to planning and execution.

After this golden hour is overcome, the tumor resection surgery enters the more controlled stage, where the general principles of neuro-oncological surgery apply intraoperative navigation, tumor biology assessment with fluorescence-guided resection when indicated and overall function-guided resection using preoperative and intraoperative mapping and monitoring. From careful planning to detailed execution, the 6–5–4 approach maximizes the changes brought by a successful MIPS surgery in neuro-oncology, providing a roadmap for an optimally personalized onco-functional balance (Figure 4).

## 6. Strengths, Limitations and Future Directions

This is a global integrated framework from MIPS in neuro-oncology, where, for the first time, a significant practical review of intraoperative challenges and the ways to overcome them are addressed. However, it lacks external validation and translation into oncological, functional and patient-centered outcomes. Despite publications from our group and others having addressed objective cannulation-related metrics such as peritubular restriction to diffusion, this framework has not been objectively evaluated. Therefore, future works should aim to assess the impact of the implementation of this framework on MIPS-related outcomes and complications. Hence, we have proposed a checklist in Table 1 to promote this validation. Also, it is important to apply this framework to different systems available on the market to understand if this is technology-specific or surgical approach-related.

## 7. Conclusions

MIPS has revolutionized the surgical treatment of deep-seated lesions in the neuro-oncology field. Preoperative structural and functional images allied to technological developments provide key information for MIPS surgical planning as supported by *the six-pillar approach* and the *five points of target trajectory complex planning.* In this paper, we focus on the intraoperative concepts that are crucial for a successful surgical delivery of the preoperative plan—(1) precannulation system checks; (2) access injury prevention; (3) tubular-tumor targeting accuracy; and (4) intracranial pressure control strategies—summarized in the *four steps for the intraoperative golden hour in MIPS for neuro-oncology*. Together, the 6–5–4 approach provides an integrated approach to the treatment of neuro-oncology patients with deep-seated lesions.

## Figures and Tables

**Table 1:** Intraoperative checklist.

**Precannulation System Check** 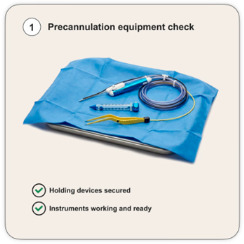	Holding devices securely placed Debulking/resecting instruments appropriately connected and tested Bipolar Ultrasonic aspirator Micro-debrider IONM systems
**Access Injury Prevention** 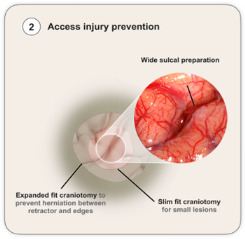	Craniotomy strategy Slim fit Expanded fit Sulcal approach Identification Preparation
**Tubular-Target Accuracy** 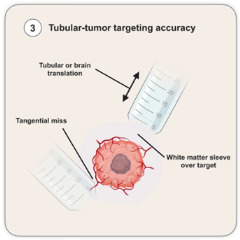	Cannulation risk assessment Translation Displacement White matter sleeve Craniotomy-cannulation mismatch Cannulation strategy Surface docking Deep docking
**ICP Management** 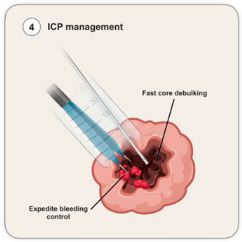	Tumor volume control Hemostasis control

IONM—Intraoperative Neuromonitoring and Mapping.

## Data Availability

The original contributions presented in this study are included in the article. Further inquiries can be directed to the corresponding author.

## Abbreviations

The following abbreviations are used in this manuscript:

MIPS	Minimally Invasive Parafascicular Surgery
ICH	Intracranial Hematoma
MRI	Magnetic Resonance Imaging
IONM	Intra-operative Neuromonitoring

## References

[1] Gassie K., Alvarado-Estrada K., Bechtle P., Chaichana K.L. (2019). Surgical management of deep-seated metastatic brain tumors using minimally invasive approaches. J. Neurol. Surg. A Cent. Eur. Neurosurg..

[2] Gassie K., Wijesekera O., Chaichana K.L. (2018). Minimally invasive tubular retractor-assisted biopsy and resection of subcortical intra-axial gliomas and other neoplasms. J. Neurosurg. Sci..

[3] Marenco-Hillembrand L., Prevatt C., Suarez-Meade P., Ruiz-Garcia H., Quinones-Hinojosa A., Chaichana K.L. (2020). Minimally invasive surgical outcomes for deep-seated brain lesions treated with different tubular retraction systems: A systematic review and meta-analysis. World Neurosurg..

[4] Taylor Z., Gupta A., Mehta N.H., Pishva S., Gupta N., Barrington N.M., Kashanian A., D’Amico R.S. (2024). Evaluating the impact of tubular retractors in glioma surgery: A systematic review and meta-analysis. Clin. Neurol. Neurosurg..

[5] Bander E.D., Jones S.H., Kovanlikaya I., Schwartz T.H. (2016). Utility of tubular retractors to minimize surgical brain injury in the removal of deep intraparenchymal lesions: A quantitative analysis of FLAIR hyperintensity and apparent diffusion coefficient maps. J. Neurosurg..

[6] Awan M., Elshalakany A., Kalaitzoglou D., Kalyal N., Sinha S., Perera A., Wroe Wright O., Gallagher M.J., Richardson D., Elhag A. (2025). Diffusion changes in minimally invasive parafascicular approach for deep-seated tumors: Impact on clinical outcomes. Neurosurg. Rev..

[7] Sinha S., Kalyal N., Gallagher M.J., Richardson D., Kalaitzoglou D., Abougamil A., Silva M., Oviedova A., Patel S., Mirallave-Pescador A. (2024). Impact of preoperative mapping and intraoperative neuromonitoring in minimally invasive parafascicular surgery for deep-seated lesions. World Neurosurg..

[8] Kelly P.J., Goerss S.J., Kall B.A. (1988). The stereotaxic retractor in computer-assisted stereotaxic microsurgery. J. Neurosurg..

[9] Pradilla G., Ratcliff J.J., Hall A.J., Saville B.R., Allen J.W., Paulon G., McGlothlin A., Lewis R.J., Fitzgerald M., Caveney A.F. (2024). Trial of early minimally invasive removal of intracerebral hemorrhage. N. Engl. J. Med..

[10] Rafati Fard A., Hibberd O., Akinduro I., Bhatti Z., Smith K.J., Patel R., Karmarkar S., Mowforth O.D., Hill C.S. (2025). Tubular retractors in neuro-oncological surgery: A systematic review and meta-analysis. Neurosurg. Rev..

[11] Kassam A.B., Labib M.A., Bafaquh M., Ghinda D., Fukui M.B., Nguyen T., Corsten M. (2015). Part II: An evaluation of an integrated systems approach using diffusion-weighted, image-guided, exoscopic-assisted, transulcal radial corridors. Innov. Neurosurg..

[12] Martucci M., Russo R., Schimperna F., D’Apolito G., Panfili M., Grimaldi A., Perna A., Ferranti A.M., Varcasia G., Giordano C. (2023). Magnetic resonance imaging of primary adult brain tumors: State of the art and future perspectives. Biomedicines.

[13] Guarnera A., Ius T., Romano A., Bagatto D., Denaro L., Aiudi D., Iacoangeli M., Palmieri M., Frati A., Santoro A. (2025). Advanced MRI, radiomics and radiogenomics in unravelling incidental glioma grading and genetic status: Where are we?. Medicina.

[14] Tuncer M.S., Salvati L.F., Grittner U., Hardt J., Schilling R., Bährend I., Silva L.L., Fekonja L.S., Faust K., Vajkoczy P. (2021). Towards a tractography-based risk stratification model for language area associated gliomas. Neuroimage Clin..

[15] Rosenstock T., Grittner U., Acker G., Schwarzer V., Kulchytska N., Vajkoczy P., Picht T. (2017). Risk stratification in motor area-related glioma surgery based on navigated transcranial magnetic stimulation data. J. Neurosurg..

[16] Sollmann N., Zhang H., Kloth C., Zimmer C., Wiestler B., Rosskopf J., Kreiser K., Schmitz B., Beer M., Krieg S.M. (2023). Modern preoperative imaging and functional mapping in patients with intracranial glioma. Rofo.

[17] Bercu M.M., Restrepo-Orozco A.F., Verhey L.H., Madura C.J., Avellino A.M., Petronio J.A., Mazaris P.A., Singer J.A. (2025). Minimally invasive interventions for intracranial pathologies using tubular retractors in the pediatric population: Safety, efficacy, technical aspects and outcomes. PLoS ONE.

[18] Sasagawa Y., Tanaka S., Kinoshita M., Nakada M. (2024). Endoscopic and exoscopic surgery for brain tumors. Int. J. Clin. Oncol..

[19] Hong C.S., Prevedello D.M., Elder J.B. (2016). Comparison of endoscope- versus microscope-assisted resection of deep-seated intracranial lesions using a minimally invasive port retractor system. J. Neurosurg..

[20] Cuellar-Hernandez J.J., Lopez-Gonzalez M.A., Olivas-Campos J.R., Tabera-Tarello P.M., Seañez-Prieto C., Eastin T.M., Song M. (2021). The use of exoscope combined with tubular retractor system for minimally invasive transsulcal resection of a ventricular atrium atypical choroid plexus papilloma: Three-dimensional operative video. Surg. Neurol. Int..

[21] Lin M., Bakhsheshian J., Strickland B., Rennert R.C., Chu R.M., Chaichana K.L., Zada G. (2020). Exoscopic resection of atrial intraventricular meningiomas using a navigation-assisted channel-based trans-sulcal approach: Case series and literature review. J. Clin. Neurosci..

[22] Lavrador J.P., Marchi F., Elhag A., Kalyal N., Mthunzi E., Awan M., Wroe-Wright O., Díaz-Baamonde A., Mirallave-Pescador A., Reisz Z. (2024). In situ light-source delivery during 5-aminolevulinic acid-guided high-grade glioma resection: Spatial, functional and oncological informed surgery. Biomedicines.

[23] De Witt Hamer P.C., Robles S.G., Zwinderman A.H., Duffau H., Berger M.S. (2012). Impact of intraoperative stimulation brain mapping on glioma surgery outcome: A meta-analysis. J. Clin. Oncol..

[24] Baig Mirza A., Vastani A., Suvarna R., Rashed S., Al-Omari A., Mthunzi E., Fayez F., Rampersad N., Jung J., Baamonde A.D. (2025). Preoperative and intraoperative neuromonitoring and mapping techniques impact oncological and functional outcomes in supratentorial function-eloquent brain tumors: A systematic review and meta-analysis. eClinicalMedicine.

[25] Gallagher M.J., Lavrador J.P., Coelho P., Mirallave-Pescador A., Bleil C., Gullan R., Ashkan K., Vergani F., Bhangoo R. (2022). Continuous microdebrider-based dynamic subcortical motor mapping: A technical advance in tubular retractor-assisted surgery. Oper. Neurosurg..

[26] Ille S., Kelm A., Schroeder A., Albers L.E., Negwer C., Butenschoen V.M., Sollmann N., Picht T., Vajkoczy P., Meyer B. (2021). Navigated repetitive transcranial magnetic stimulation improves the outcome of postsurgical paresis in glioma patients: A randomized, double-blinded trial. Brain Stimul..

[27] Darrigues E., Elberson B.W., De Loose A., Lee M.P., Green E., Benton A.M., Sink L.G., Scott H., Gokden M., Day J.D. (2021). Brain tumor biobank development for precision medicine: Role of the neurosurgeon. Front. Oncol..

[28] Wang J.H., Xia J., Wu P.F., Yi L., Yu S.C. (2025). An optimized tissue sampling scheme guided by MRI features reveals intratumoral heterogeneity in glioblastoma. Sci. Rep..

[29] Oh Y.T., Cho H.J., Kim J., Lee J.H., Rho K., Seo Y.J., Choi Y.S., Jung H.J., Song H.S., Kong D.S. (2014). Translational validation of personalized treatment strategy based on genetic characteristics of glioblastoma. PLoS ONE.

[30] Ivren M., Grittner U., Khakhar R., Belotti F., Schneider H., Pöser P., D’Agata F., Spena G., Vajkoczy P., Picht T. (2023). Comparison of anatomical-based vs. nTMS-based risk stratification model for predicting postoperative motor outcome and extent of resection in brain tumor surgery. Neuroimage Clin..

[31] Sollmann N., Kelm A., Ille S., Schröder A., Zimmer C., Ringel F., Meyer B., Krieg S.M. (2018). Setup presentation and clinical outcome analysis of treating highly language-eloquent gliomas via preoperative navigated transcranial magnetic stimulation and tractography. Neurosurg. Focus.

[32] Raffa G., Quattropani M.C., Scibilia A., Conti A., Angileri F.F., Esposito F., Sindorio C., Cardali S.M., Germanò A., Tomasello F. (2018). Surgery of language-eloquent tumors in patients not eligible for awake surgery: The impact of a protocol based on navigated transcranial magnetic stimulation on presurgical planning and language outcome, with evidence of tumor-induced intra-hemispheric plasticity. Clin. Neurol. Neurosurg..

[33] Kram L., Schroeder A., Meyer B., Krieg S.M., Ille S. (2024). Function-guided differences of arcuate fascicle and inferior fronto-occipital fascicle tractography as diagnostic indicators for surgical risk stratification. Brain Struct. Funct..

[34] Sollmann N., Fratini A., Zhang H., Zimmer C., Meyer B., Krieg S.M. (2020). Associations between clinical outcome and tractography based on navigated transcranial magnetic stimulation in patients with language-eloquent brain lesions. J. Neurosurg..

[35] Raffa G., Marzano G., Curcio A., Espahbodinea S., Germanò A., Angileri F.F. (2022). Personalized surgery of brain tumors in language areas: The role of preoperative brain mapping in patients not eligible for awake surgery. Neurosurg. Focus.

[36] Feng J.J., Cheok S.K., Shiroishi M.S., Zada G. (2023). Tumor characteristics guiding selection of channel-based versus open microscopic approaches for resection of atrial intraventricular meningiomas: Patient series. J. Neurosurg. Case Lessons.

[37] Dannhoff G., Poudel P.P., Bhattarai C., Kalthur S.G., Maldonado I.L. (2023). Depicting the anatomy of the gyral white matter: Ubi sumus? quo vadimus?. Brain Commun..

[38] Umana G.E., Scalia G., Graziano F., Maugeri R., Alberio N., Barone F., Crea A., Fagone S., Giammalva G.R., Brunasso L. (2021). Navigated transcranial magnetic stimulation motor mapping usefulness in the surgical management of patients affected by brain tumors in eloquent areas: A systematic review and meta-analysis. Front. Neurol..

[39] Bennett C., González M., Tapia G., Riveros R., Torres F., Loyola N., Veloz A., Chabert S. (2022). Cortical mapping in glioma surgery: Correlation of fMRI and direct electrical stimulation with Human Connectome Project parcellations. Neurosurg. Focus.

[40] Rajashekar D., Lavrador J.P., Ghimire P., Keeble H., Harris L., Pereira N., Patel S., Beyh A., Gullan R., Ashkan K. (2022). Simultaneous motor and visual intraoperative neuromonitoring in asleep parietal lobe surgery: Dual strip technique. J. Pers. Med..

[41] Genel O., Price S., Marchi F., Elhag A., WroeWright O., Mirallave-Pescador A., Bibby S., Ashkan K., Vergani F., Bhangoo R. (2024). O-ARM navigation in tubular retractor-assisted minimal invasive parafascicular approach: Technical note. J. Surg. Case Rep..

[42] Capitanio J.F., Donofrio C.A., Panni P., Barzaghi L.R., Bailo M., Gagliardi F., Mortini P. (2021). Microsurgical endoportal MRI/US-navigated approach for the resection of large intraventricular tumors: A 20-consecutive patients case series. Br. J. Neurosurg..

